# Supplementary material for: Transformation of Amorphous Terbium Metal–Organic Framework on Terbium Oxide TbOx(111) Thin Film on Pt(111) Substrate: Structure of TbxOy Film
Source: Nanomaterials (Basel). 2022 Aug 17;12(16):2817. doi: 10.3390/nano12162817 (PMC9412416; doi:10.3390/nano12162817)
Supplement: Supplementary file 1 [file nanomaterials-12-02817-s001.zip › nanomaterials-1868201-supplementary.pdf]

nanomaterials

MDPI

Supplementary Material

Transformation of Amorphous Terbium Metal–Organic Framework on Terbium Oxide $TbO_x(111)$ Thin Film on Pt(111) Substrate: Structure of Tb_xO_y Film

Helena Brunckova [1,*], Erika Mudra [1], Magdalena Streckova [1], Lubomir Medvecky [1], Tibor Sopcak [1], Ivan Shepa [1], Alexandra Kovalcikova [1], Maksym Lisnichuk [1] and Hristo Kolev [2]

[1] Institute of Materials Research, Slovak Academy of Sciences, Watsonova 47, 040 01 Kosice, Slovakia
[2] Institute of Catalysis, Bulgarian Academy of Sciences, Acad. G. Bonchev St., BU-1113 Sofia, Bulgaria
* Correspondence: hbrunckova@saske.sk; Tel.: +421-55-7922-455

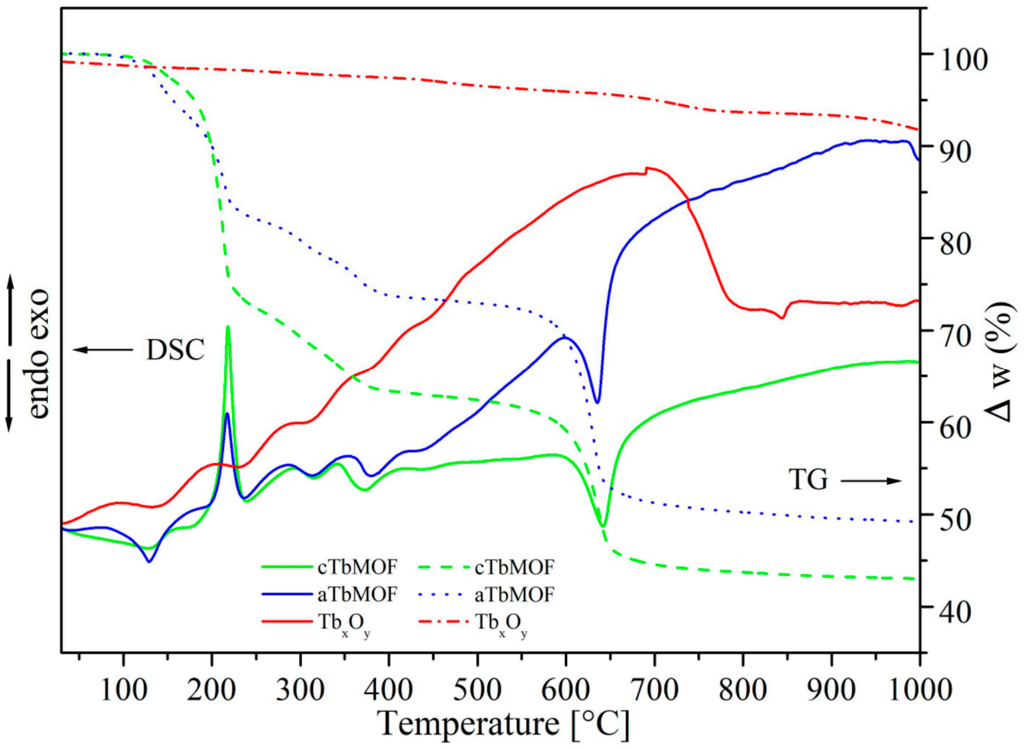

Figure S1. DSC/TG curves of crystalline (cTbMOF), amorphous (aTbMOF), and amorphous aTbMOF, heated at 450 °C (Tb_xO_y) powders.

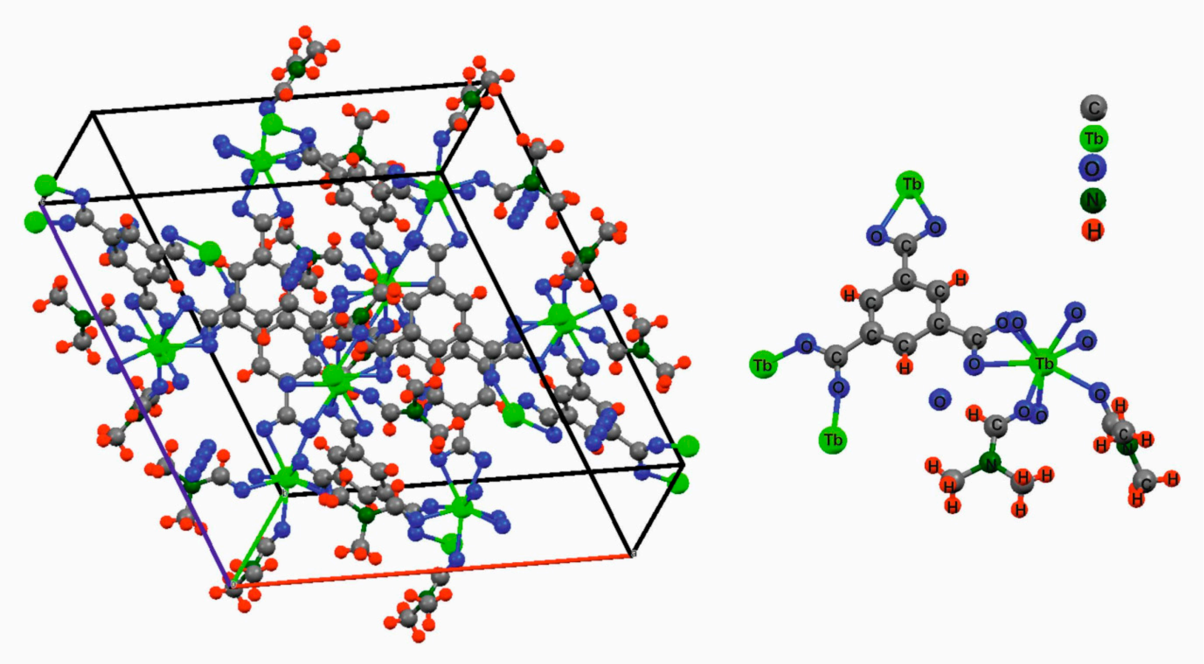

Figure S2. Coordination environment of Tb(BTC)(DMF)$_2$(H_2O); TbMOF.

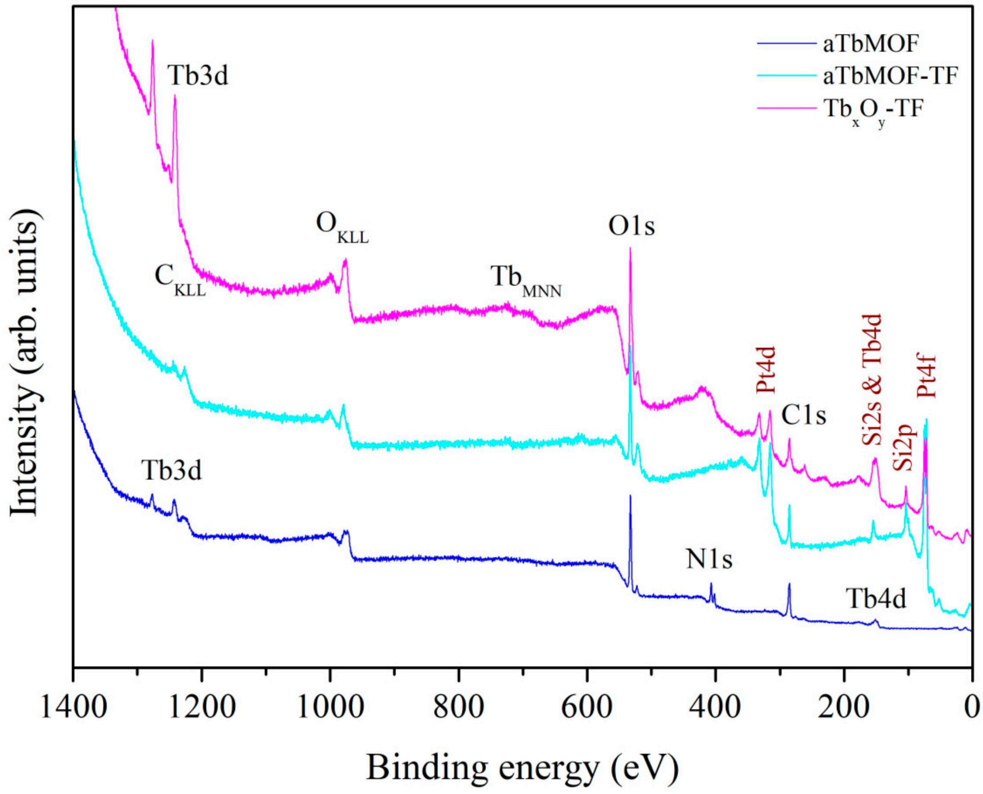

Figure S3. XPS survey spectra of amorphous powder (aTbMOF), amorphous thin film (aTbMOF-TF), and Tb_xO_y-TF on SiO_2/Si substrates with Pt interlayer.

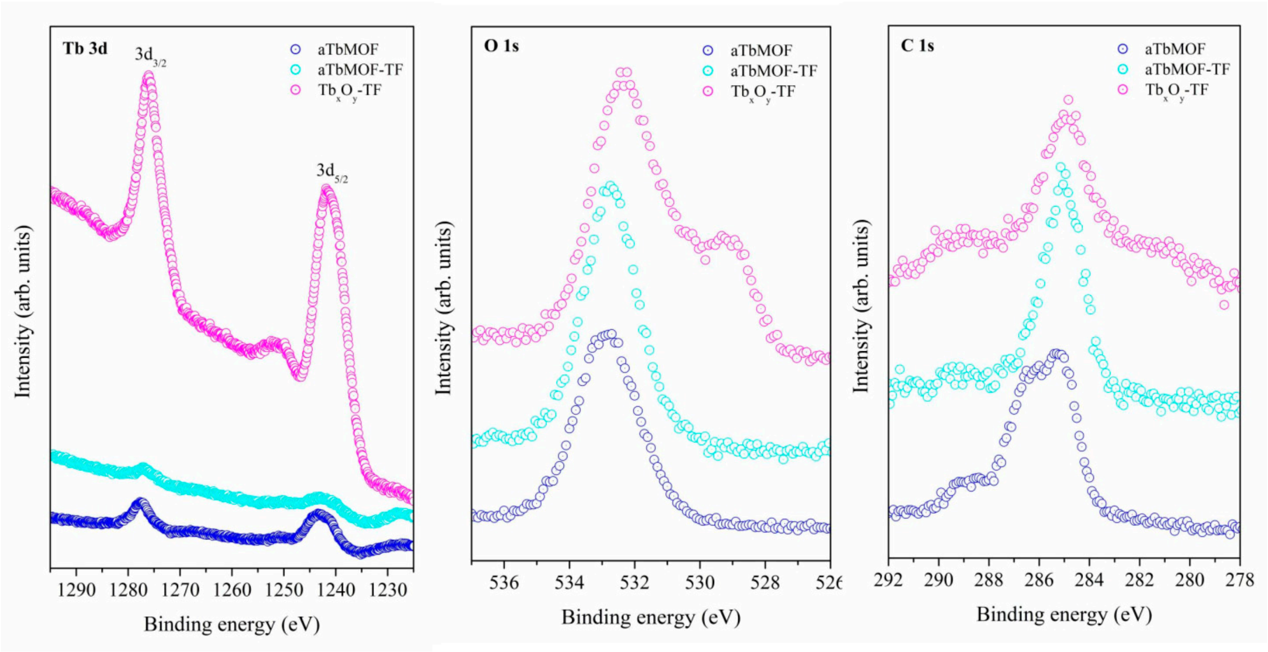

Figure S4. HR XPS spectra of Tb 3d, O 1s, and C 1s for amorphous aTbMOF powder and aTbMOF-TF film and Tb_xO_y-TF film.

Table S1. XPS elemental atomic % of aTbMOF, aTbMOF-TF and Tb_xO_y-TF samples.

Sample	Tb	O	C	N	Pt	Si
aTbMOF	2.00	35.76	45.23	17.00	-	-
aTbMOF-TF	0.56	30.22	30.25	-	20.37	18.61
Tb_xO_y-TF	9.87	44.34	27.29	-	6.61	11.89

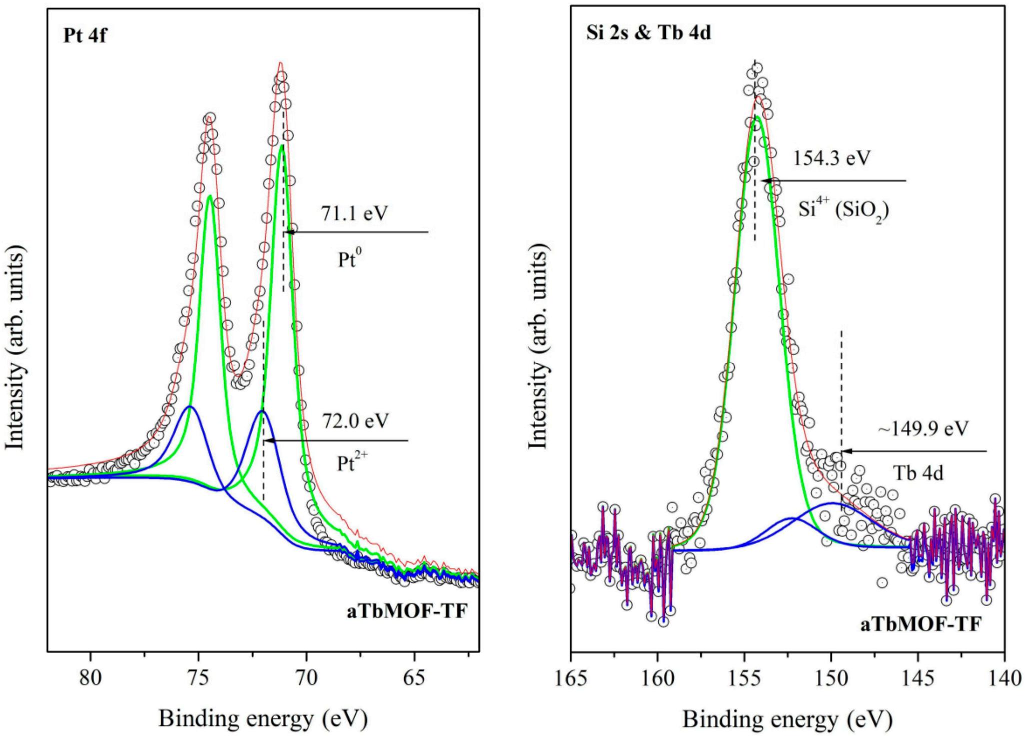

Figure S5. HR XPS spectra of Pt 4f and Si 2s & Tb 4d for amorphous aTbMOF-TF thin film.

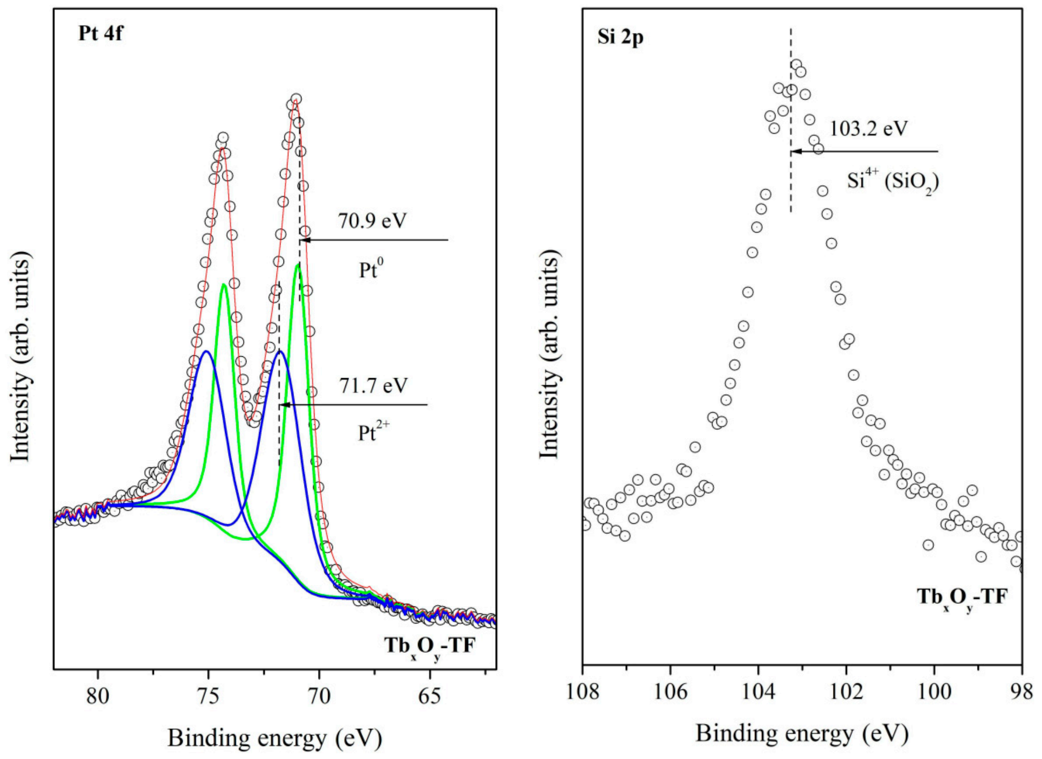

Figure S6. HR XPS spectra of Pt 4f and Si 2p (substrate) for Tb_xO_y-TF thin film.

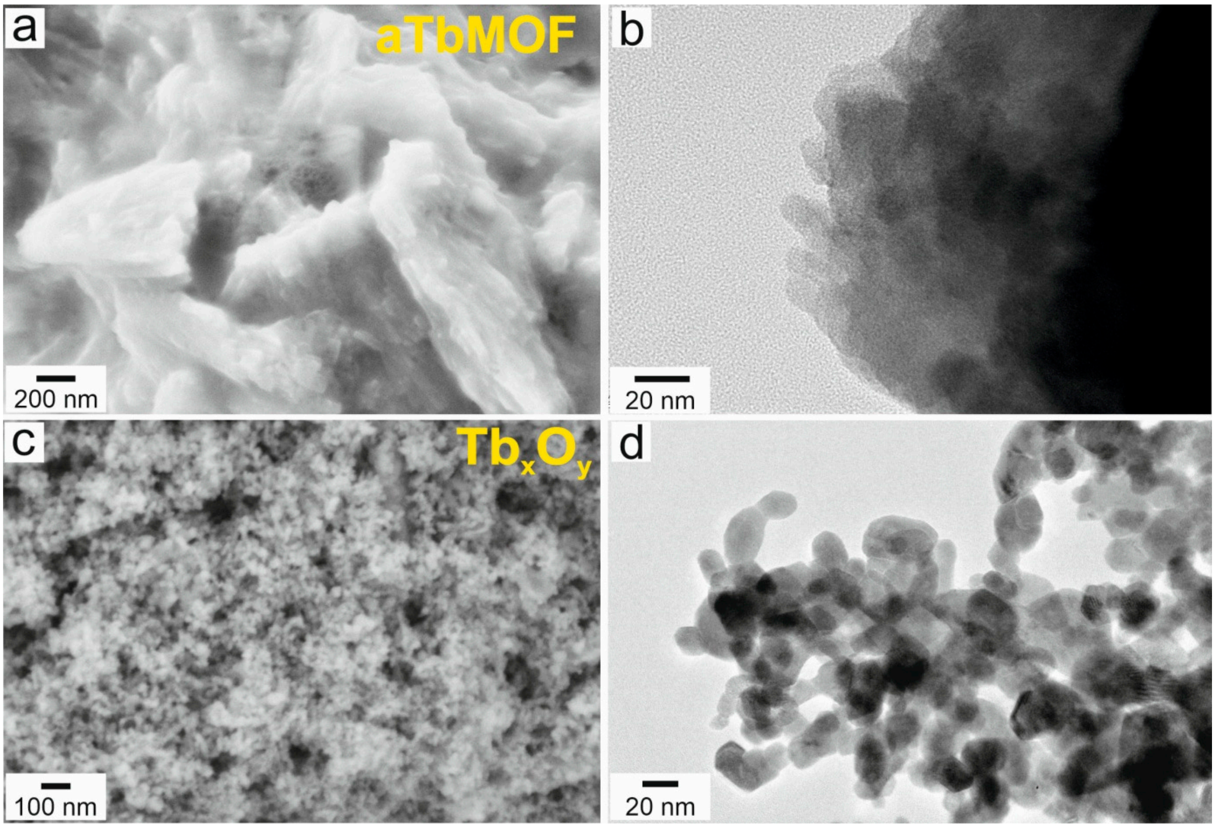

Figure S7. SEM and TEM images of (**a**,**b**) amorphous aTbMOF and (**c**,**d**) Tb_xO_y powders.

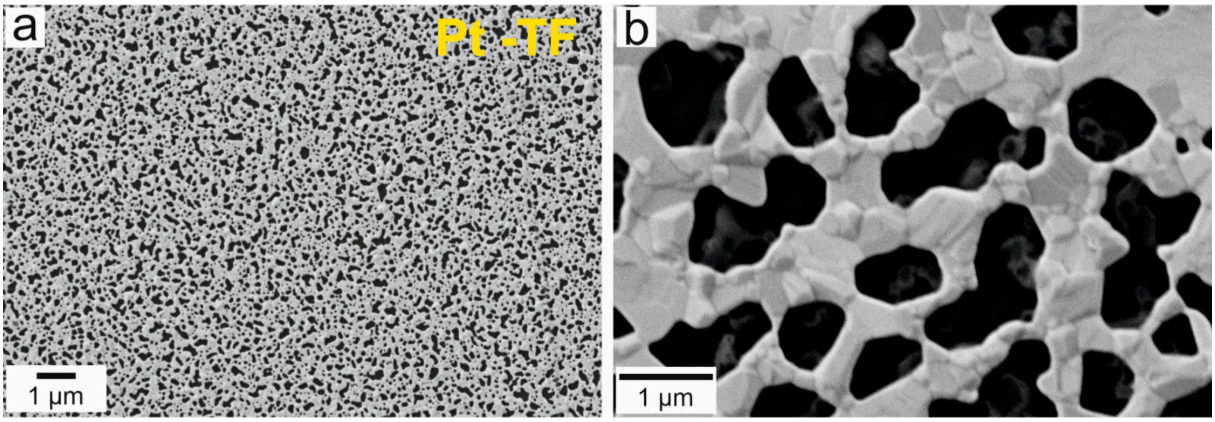

Figure S8. SEM microstructures of Pt/SiO_2/Si substrate sample (**a**) as-deposited and (**b**) annealed at 450 °C.

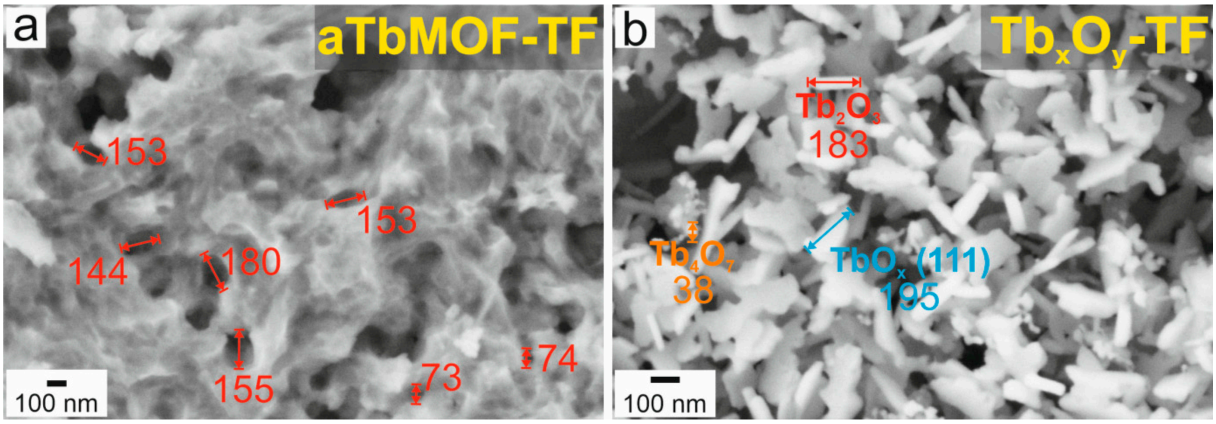

Figure S9. SEM surface microstructures of (**a**) aTbMOF-TF (with pore sizes) and (**b**) Tb_xO_y-TF thin films (with particle sizes).

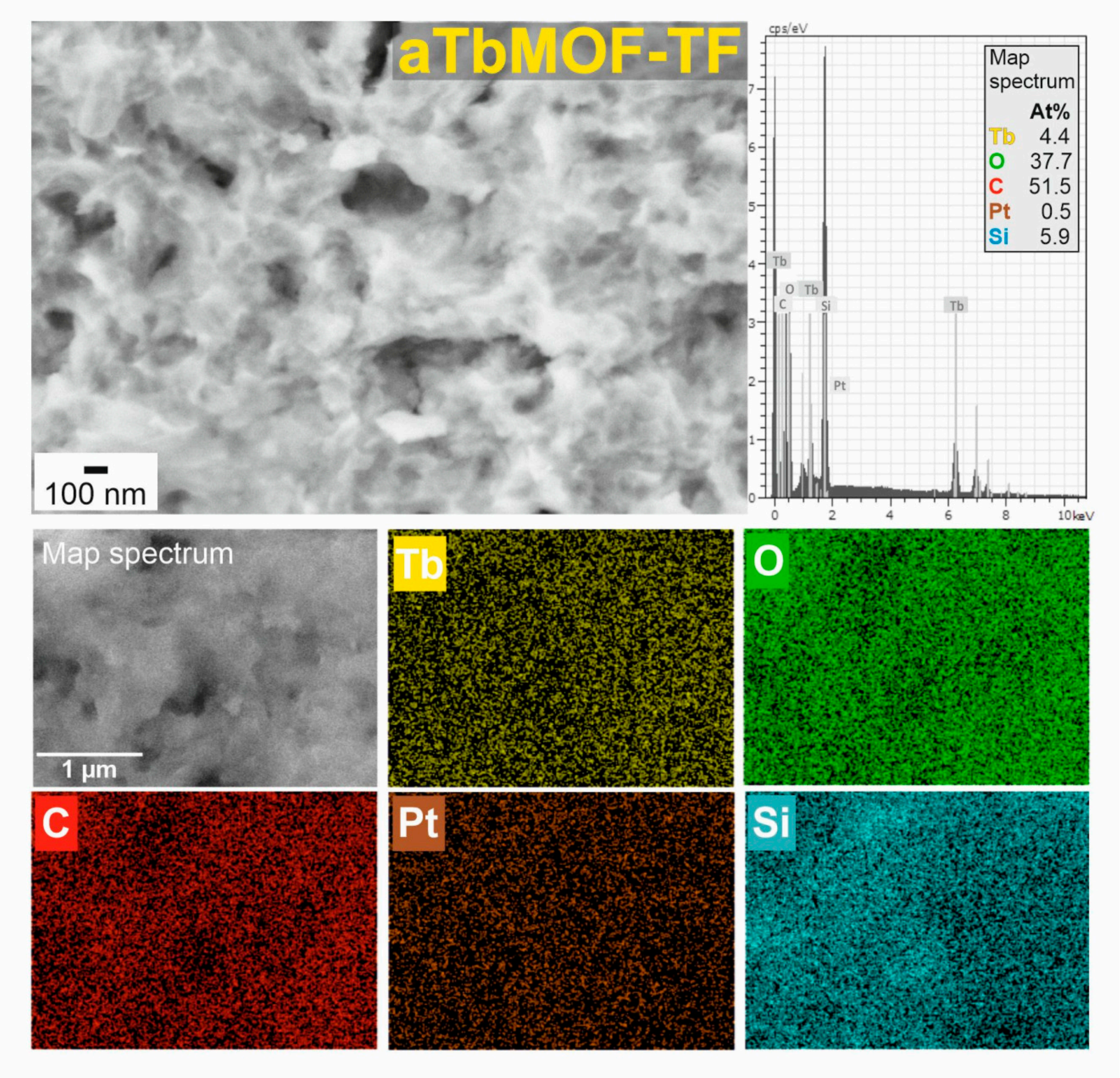

Figure S10. SEM surface microstructures and EDS elemental mapping of the amorphous aTbMOF-TF thin film.

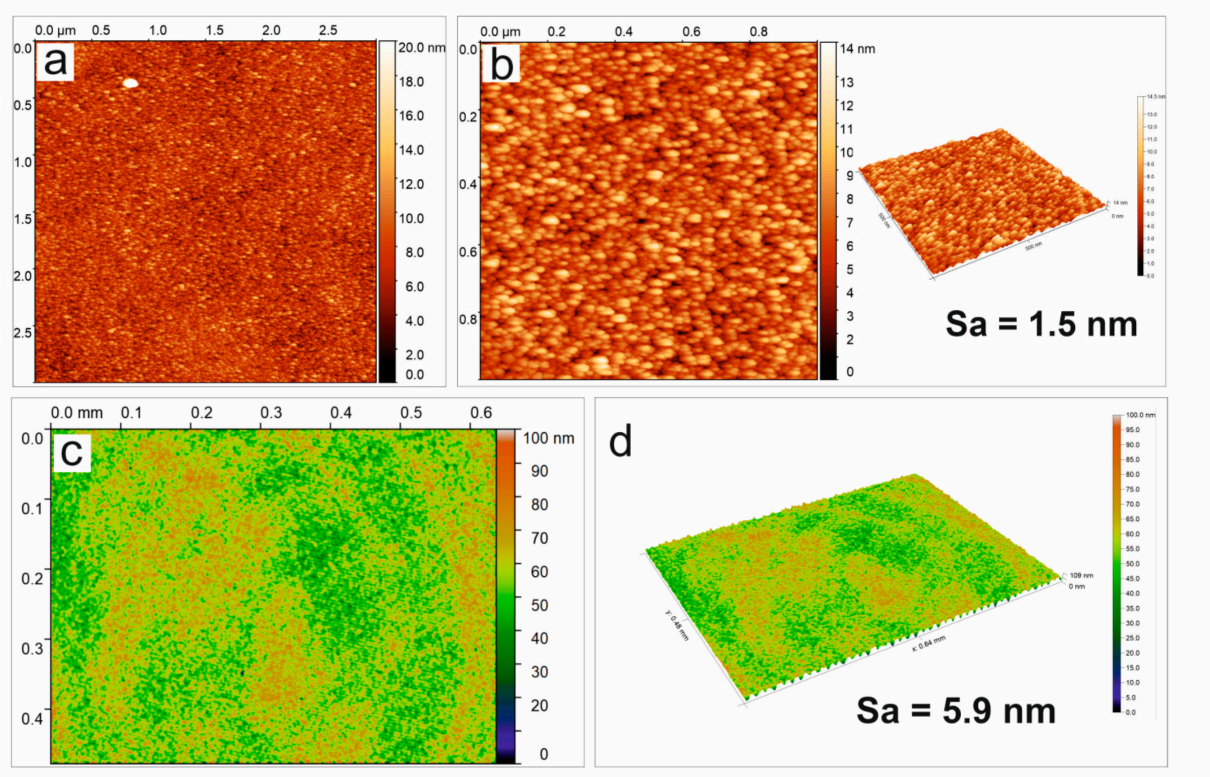

Figure S11. (**a**,**b**) 2D and 3D-AFM topography and (**c**,**d**) Confocal microscopy (CM) scans of the Pt/SiO_2/Si substrate sample in isometric and 3D representations of Pt-interlayer.
